# Why so Many of Us Fail, Both as Dentists and Business Men

**Published:** 1914-10-15

**Authors:** W. H. Chilton

**Affiliations:** Clayton, N. Mex.


### WHY SO MANY OF US FAIL, BOTH AS DENTISTS AND BUSINESS MEN.

W. H. CHILTON, D.D.S., CLAYTON, N. MEX.

Now to begin with: I wish to make a short explanation in regard to this subject and heading, and to explain that this article is not written to find fault, or call your attention to these things simply to be making a kick in a pessimistic mood, but because twenty-five years of close study of the question has taught me that this is one of, if not the greatest question that we have to contend with today. And still the answer is very simple and easy to understand, when we once get down to brass tacks and look for the trouble.

Now some of you, especially you younger men, may say: "But so many of us do not fail; we make a living out of it and so make good." And right there is the rub; because you have simply made a living or even saved a little money it does not therefore follow that you have been a success. You must not only do that, but in the meantime you should make a name for yourself both as a dentist and a man; and unless your work has been such as to gather around you the best people of the community who believe in you and your work, and you have educated them to what dentistry really means, and taught them the real value of good work and the utter worthlessness of poor work, and they really and honestly believe that you are the best dentist in the world, you can really say that you have been a success.

The whole trouble is that too many of us are dead but don't know it, and are only walking around to save funeral expenses. While we breath and walk and sometimes talk as though we were living beings, still we are not alive and wide-awake as we should be, with our whole heart in the work, striving with might and main to make every operation perfect. Too many of us are inclined when we get hold of a mean, hard job, especially if we are not getting as much money out of it as we should, to say, or rather think, "Oh, well, that is not just right, but it is good enough." Now in this connection I wish to say, and say with all the power that lies within me, so that you may not forget, that this thought causes more failures and ruins more promising young men than any other one thing, not only in our business, but in every occupation that we may enter.

No operation, however simple, is good enough unless we have given to it our whole attention, giving to our patient the very best that lies within us, and being able to say that it is as perfect as it is possible for us to make it. Anything short of this is both wrong and dishonest. An injustice to your patients, a crime against our profession, and, worst of all, you are proving untrue to yourself.

Too many of us conduct our business in a slipshod manner, without stopping to use the brain that God gave us, and as soon as we get to the point where we can make a bare living and not be afraid of being sued for malpractice, we consider we know it all, and think that we can conduct our business without giving it our best efforts, and in so doing get into a rut which we find it impossible to climb out of after we have followed it for a few years.

Now each and every one of you is saying to himself, "Yes that is true about some of us, but I don't do that way." Of course I know that none of us ever did that way, because it is the other fellow that always does these things, but I am talking to the other fellow, and to illustrate my point will tell about a case I had a year or two ago: A prominent judge of our state came to me with an aching tooth, stating it had kept him awake all night, and, pointing to a lower first bicuspid, which was broken off almost to the gum line, said, "That is the tooth and I have been having it treated for three months, but still every week or so it has a spell of aching."

Upon examination I found a good, healthy root, with absolutely no soreness, and with a perfectly filled canal. A moment's thought told me that it was impossible for that tooth to have hurt all night and not be sore to the touch in the morning, and the following conversation took place: "Judge, that is not the tooth that kept you awake." "Oh, yes, it is, for I have had it treated by a good dentist for three months; one whom I know is good and has worked for me nine years and I know it is the tooth." "Well, Judge, it makes no difference how good your dentist is, or even if he has worked for you ninety-nine years, it is impossible for that to be the tooth." I then went on to explain to him fully just why that particular tooth could not possibly have caused the trouble, and upon examination found an exposed nerve between the first and second molar. I cleaned it out and within two minutes after I had applied medicine his tooth was easy.

I then told him to come to me if the pain returned and I would treat it and keep it easy until he could get back home, and then he should go to his dentist and have him kill the nerve and fill it, also to put a crown on the tooth he had treated for three months, as it had been ready for it for at least two months. He then said, "But can't you do the work while I am here?" I told him, "Yes I could do it, but your home dentist who has had charge of your mouth for nine years knows better than I do the peculiarities of your teeth, besides the place to have your dental work done is at home, where you can see your dentist at regular intervals and have your mouth kept in condition."

Upon my advice he did this, and when he came to hold his next court, six months later, the first thing he said when we met was, "You were right about that tooth and he did as you said."

When I eased his tooth and he asked me what my bill was, I told him, "Not a cent. I have only eased you temporarily and am willing to do this much to help a brother dentist, knowing he would do as much under the same circumstances for me." In doing this I lost a dollar in ready money, or the value of twenty beers or a dozen trout flies, but I gained tenfold its value in the knowledge of treating a brother dentist as I should want him to treat me and gained a lifetime friend.

Now, perhaps this dentist was a good man and a fine dentist, but he had got in the rut and without thinking, or taking the trouble to use his brains, treated the tooth that he was told had caused the trouble, and in so doing made a perfect flat.

Case Number Two. A young doctor came to me and said that he kept his teeth in good condition and had them examined every year, but had a little trouble between two of his molars. However, there could not be much wrong, as he had had his teeth examined twice within the year by good dentists. Upon examination I found seven cavities, two of which were almost to the nerve which had to be killed.

Now, can any of you say that those two dentists were alive or awake to the interests of either their patients or themselves?

Case Number Three. A lady who left Clayton and moved to Holly, Colorado, some seven years ago, but who came back once a year to have her own and the children's teeth kept in shape, brought her little girl, some eight years old, to me to have my advice about the work she was having done by the Holly dentists. This work consisted of quite a long, hard job in orthodontia, and one tooth had to be turned almost half around. Upon examination I found that, while the bands and wire looked in the mouth something like the pictures we see in our dental books, still he had been asleep and in tightening the screws was turning the tooth in the wrong direction and in another month or two the child's mouth would have been ruined. Although they had paid fifty dollars down on this work, upon my advice she had it all torn out and went to another dentist and had the work done.

Now, no doubt this dentist was honest and wanted to do right, but would not take the trouble to wake up and do a little thinking on his own account and so made a flat failure.

I could go on and give case after case showing what a large proportion of us get into this rut, but will close with one other illustration.

I may be just a little nutty upon the subject of pyorrhea and prophylaxis, but for eighteen years I have talked it to every patient that came into my office; have explained fully to them that unless they keep their mouths clean and pure that they could not expect good health, and have impressed on them in every way possible the importance of getting their mouths in a sanitary and healthy condition. This has been one of my hobbies and I talk it morning, noon and night. Well, fully seven thousand people have located in Union County in the past six years and these people came from every section of the country, almost every family having a different dentist before coming out here. In the past six years, I have treated fully five hundred of these newcomers for pyorrhea, many of them in very bad condition, and while most of them had a lot of filling and bridge work in their mouths and had spent quite a lot of money in trying to save their teeth, only two out of the five hundred had ever been told even how to wash their teeth, and not a word said about pyorrhea or its cure. Dozens of them said, when I told them their teeth were past saving and that they would lose them in a very few years in spite of all we could now do, as it was too late to save them, "But, Doctor, my dentist was a fine one and would have told me if this had been so before I came out here, and surely it has started since I came. He would not treat me that way I know. You must be mistaken about when it started."

Now, is this not absolutely pitiful? That out of five hundred of us scattered all over the United States, only two were alive and did their duty to their patients? And with such a vast majority of us "Asleep at the switch," is it any wonder that we fail, both as dentists and as business men?

Sometime ago I noticed in one of the journals where a dentist perhaps fifty years old had written to the editor with a whine stating his troubles, and asking for advice complaining that the young men were pushing the older ones to the wall. That the younger people had all deserted him and that he could no longer make a living working for the older ones. Now, while this is a sad state of affairs, especially for him, it is absolutely his own fault, and if he had only been honest with himself and looked in earnest for the real cause of this desertion, he would have found that it was all his own fault and he had got so deep in the rut that he forced his patients to seek another dentist who was wide-awake. The man who keeps alive need never fear an end like this, and with me if the time ever comes that even a very small per cent of my patients desert for either a younger, older, uglier or better looking man than I am, I will realize that I have gone dead and lost interest in my profession and that it is time for me to quit and go to digging ditches or some other job that I can follow and make a success without using very much gray matter.

In conclusion I will say, the one great secret of success, not only in dentistry, but in every other walk of life, lies in eternal vigilance, always giving our best efforts, and never under any circumstances slighting our work, even in the smallest degree.

Hoping that this paper may help at least one brother dentist in attaining success in his chosen profession, I leave it with you for dissection.—*Western Journal.*

